# Supplementary material for: Bacterial protein domains with a novel Ig‐like fold target human CEACAM receptors
Source: EMBO J. 2021 Feb 1;40(7):e106103. doi: 10.15252/embj.2020106103 (PMC8013792; doi:10.15252/embj.2020106103)
Supplement: Supplementary file 1 — Appendix [file EMBJ-40-e106103-s005.pdf]

Table of Contents

1. **Appendix Figure S1:** CEACAM1 does not bind to *S. sanguinis.*
2. **Appendix Figure S2:** The N-terminal domain of CEACAM1 binds β-IgSF.
3. **Appendix Figure S3:** Binding of CEACAMs to GBS.
4. **Appendix Figure S4:** Alignment of IgI3 domain sequences from β protein of GBS
5. **Appendix Figure S5:** Predicted structures of IgI3 homologs.
6. **Appendix Figure S6:** IgI3 domains bind CEACAM1 through alternative binding pockets.
7. **Appendix Figure S7:** Isothermal titration calorimetry (ITC) binding curve of CEACAM1-N and R28-IgI3.
8. **Appendix Table S1.** Bacterial strains used in this study.
9. **Appendix Table S2:** Expression vectors constructs.
10. **Appendix Table S3:** Isothermal Titration Calorimetry (ITC) binding curves constants and thermodynamic paramters for CEACAM-N and β-IgSF interactions.
11. **Appendix Table S4**. Data collection and refinement statistics for β-IgI3 and CEACAM1 (CC1)-N.
12. **Appendix Table S5.** Contact sites in (β-IgI3)(CEACAM1-N) complex.
13. **Appendix Table S6:** Isothermal Titration Calorimetry (ITC) binding curves constants and thermodynamic paramters for CEACAM1 (CC1)-N and β-IgI3 mutants.
14. **Appendix Table S7:** Cell lines used in this study.
15. **Appendix Table S8:** Antibodies used in this study.

A

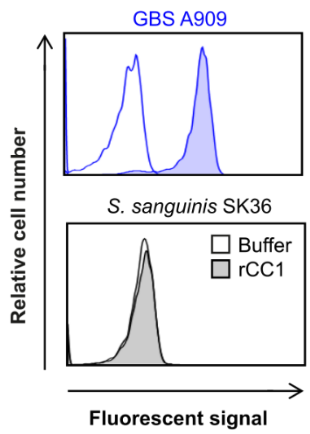

Appendix Figure S1: CEACAM1 does not bind to *S. sanguinis.* Binding of rCEACAM1-His (rCC1) (10 µg/mL) to GBS strain A909 and *S. sanguinis* strain SK36. Fluorescence of bacteria was measured by flow cytometry. Data representative of *n* = 3.

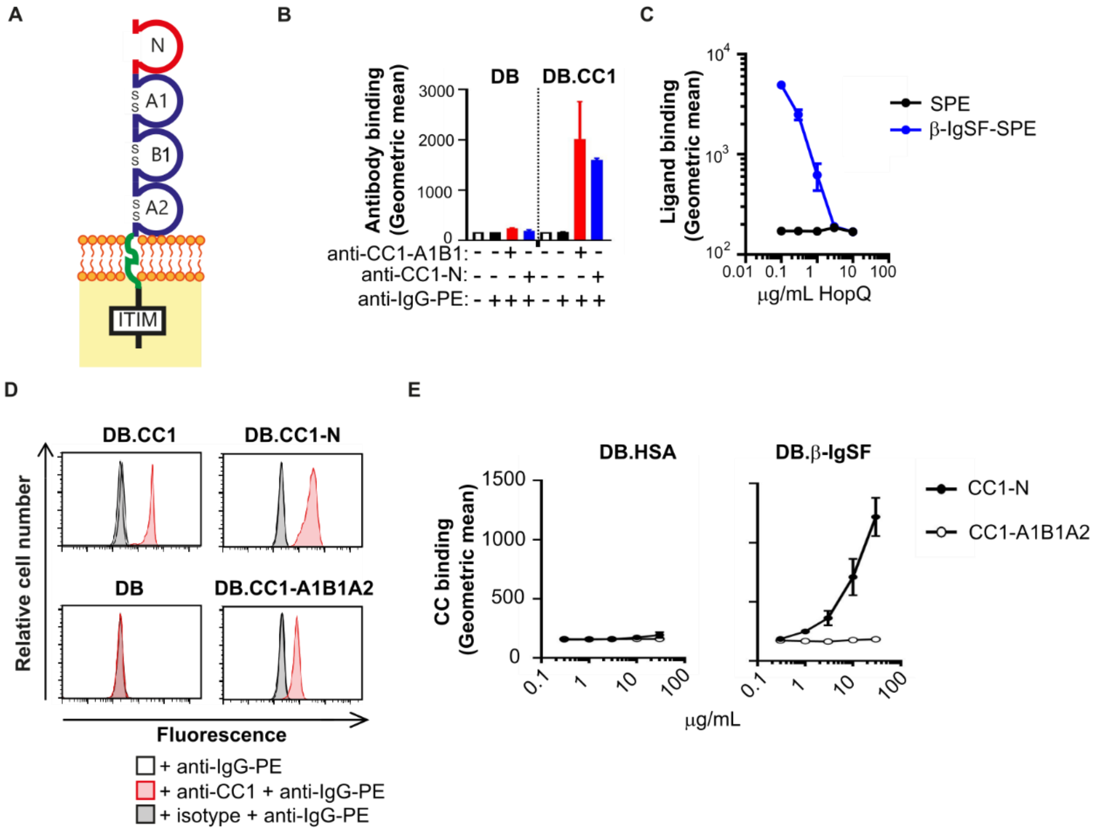

Appendix Figure S2: The N-terminal domain of CEACAM1 binds β-IgSF. A) Schematic of CEACAM1-4L (CC1) structure. The extracellular region of the receptor is composed of the N terminal (IgV-like) domain, and the A1, B1 and A2 (IgC2) domains. The cytoplasmic tail of full length CEACAM1 contain immunoreceptor tyrosine-based inhibitory motifs (ITIM) for signalling. **B)** Control showing binding of anti-CC1-N or anti-CC1-A1B1 mAb (5 μg/mL) to dynabeads (DB) coated with CC1 or buffer. Mean and SD values are reported for n = 3. **C)** rHopQ inhibits binding of β-IgSF tetramers (3 μg/mL) to DB.CC1. **D)** Control showing binding of anti-CC1 mAb to DB coated with rCC1, rCC1-N and rCC1-A1B1A2 but not to controls. Data representative of n = 3. **E)** rCC1-N but not rCC1-A1B1A2 binds to β-IgSF-, but not HSA-, coated DB in a concentration-dependent manner. Mean and SD values are reported for n = 3. Fluorescence of DB in B, C, D and E was measured by flow cytometry. Note: error bars in controls are smaller than symbols.

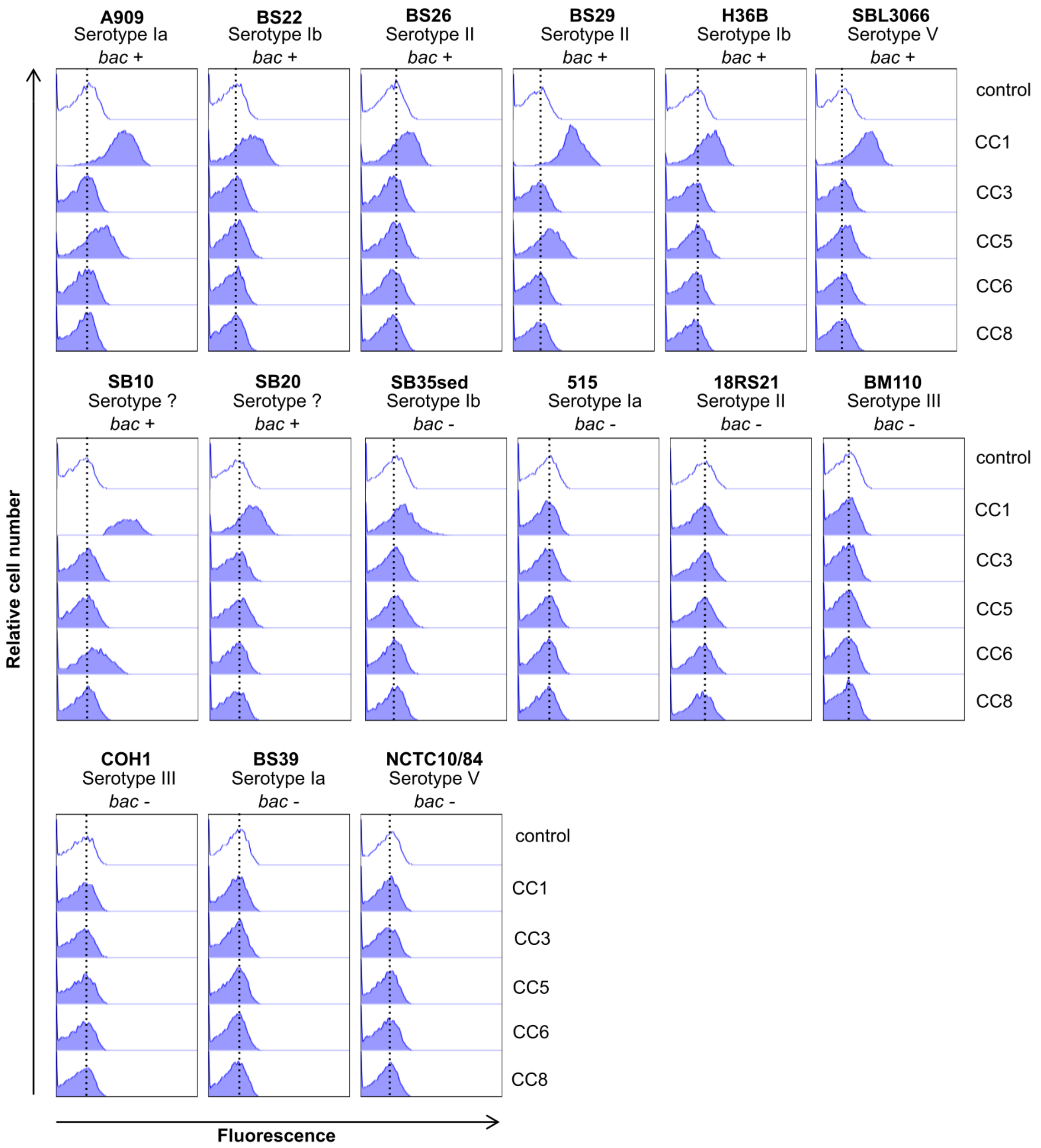

Appendix Figure S3: Binding of CEACAMs to GBS. Binding rCEACAM1-His (CC1), rCEACAM3-His (CC3), rCEACAM5-His (CC5), rCEACAM6-His (CC6) and rCEACAM8-His (CC8) (10 µg/mL) to a panel of GBS strains. Serotype and carriage of *bac* gene is indicated for each strain. Data representative of *n* = 3. Fluorescence of bacteria was measured by flow cytometry.

```
                                    10        20        30        40
EPU95478.1:428540/1-113        ANENNQQKIELTVSPENITVYEGEDVKFTVTAKSDSKTTLDFSDL
WP_000295505.1:94206/1-113     ANENNQQKIELTVSPENITVYEGEDVKFTVTAKSDSKTTLDFSDL
KXA56917.1:93205/1-113         ANENNQQKIELTVSPENITVYEGEDVKFTVTAKSDSKTTLDFSDL
WP_000477137.1:428540/1-113    ANENNQQKIELTVSPENITVYEGEDVKFTVTAKSDSKTTLDFSDL
WP_017650912.1:428540/1-113    ANENNQQKIELTVSPENITVYEGEDVKFTVTAKSDSKTTLDFSDL
WP_132935284.1:428540/1-113    ANENNQQKIELTVSPENITVYEGEDVKFTVTAKSDSKTTLDFSDL
WP_001933343.1:428540/1-113    ANENNQQKIELTVSPENITVYEGEDVKFTVTAKSDSKTTLDFSDL
WP_025195781.1:428540/1-113    ANENNQQKIELTVSPENITVYEGEDVKFTVTAKSDSKTTLDFSDL
WP_132868240.1:428540/1-113    ANENNQQKIELTVSPENITVYEGEDVKFTVTAKSDSKTTLDFSDL
WP_041980967.1:428540/1-113    ANENNQQKIELTVSPENITVYEGEDVKFTVTAKSDSKTTLDFSDL
CFQ92576.1:130/1-113           ANENNQQKIELTVSPENITVYEGEDVKFTVTAKSDSKTTLDFSDL
EPV41091.1:160272/1-113        ANENNQQKIELTVSPENITVYEGEDVKFTVTAKSDSKTTLDFSDL
WP_049458979.1:428540/1-113    ANENNQQKIELTVSPENITVYEGEDVKFTVTAKSDSKTTLDFSDL
SQA16054.1:253365/1-113        ANENNQQKIELTVSPENITVYEGEDVKFTVTAKSDSKTTLDFSDL
BAE45252.1:428540/1-113        ANENNQQKIELTVSPENITVYEGEDVKFTVTAKSDSKTTLDFSDL
WP_055345127.1:428540/1-113    ANENNQQKIELTVSPENITVYEGEDVKFTVTAKSDSKTTLDFSDL
WP_017650863.1:428540/1-113    ANENNQQKIELTVSPENITVYEGEDVKFTVTAKSDSKTTLDFSDL
CNE84135.1:307419/1-113        ANENNQQKIELTVSPENITVYEGEDVKFTVTAKSDSKTTLDFSDL
WP_060457561.1:428540/1-113    ANENNQQKIELTVSPENITVYEGEDVKFTVTAKSDSKTTLDFSDL
WP_060458554.1:428540/1-113    ANENNQQKIELTVSPENITVYEGEDVKFTVTAKSDSKTTLDFSDL
WP_000477135.1:428540/1-113    ANENNQQKIELTVSPENITVYEGEDVKFTVTAKSDSKTTLDFSDL
WP_121070430.1:428540/1-113    ANENNQQKIELTVSPENITVYEGEDVKFTVTAKSDSKTTLDFSDL
P27951.1:428540/1-113          ANENNQQKIELTVSPENITVYEGEDVKFTVTAKSDSKTTLDFSDL
WP_047199360.1:428540/1-113    ANENNQQKIELTVSPENITVYEGEDVKFTVTAKSDSKTTLDFSDL
WP_017647376.1:428540/1-113    ANENNQQKIELTVSPENITVYEGEDVKFTVTAKSDSKTTLDFSDL
WP_141445744.1:401513/1-113    ANENNQQKIELTVSPENITVYEGEDVKFTVTAKSDSKTTLDFSDL
WP_050152779.1:428540/1-113    ANENNQQKIELTVSPENITVYEGEDVKFTVTAKSDSKTTLDFSDL
WP_000477136.1:428540/1-113    ANENNQQKIELTVSPENITVYEGEDVKFTVTAKSDSKTTLDFSDL
EPV99179.1:351463/1-113        ANENNQQKIELTVSPENITVYEGEDVKFTVTAKSDSKTTLDFSDL
WP_071150949.1:419531/1-113    ANENNQQKIELTVSPENITVYEGEDVKFTVTAKSDSKTTLDFSDL
WP_000477141.1:428540/1-113    ANENNQQKIELTVSPENITVYEGEDVKFTVTAKSDSKTTLDFSDL
WP_157140373.1:377489/1-113    ANENNQQKIELTVSPENITVYEGEDVKFTVTAKSDSKTTLDFSDL
WP_000477134.1:428540/1-113    ANENNQQKIELTVSPENITVYEGEDVKFTVTAKSDSKTTLDFSDL
WP_094754263.1:428540/1-113    ANENNQQKIELTVSPENITVYEGEDVKFTVTAKSDSKTTLDFSDL
WP_001905993.1:428540/1-113    ANENNQQKIELTVSPENITVYEGEDVKFTVTAKSDSKTTLDFSDL
WP_017645156.1:423535/1-113    ANENNQQKIELTVSPENITVYEGEDVKFTVTAKSDSKTTLDFSDL
WP_094969800.1:428540/1-113    ANENNQQKIELTVSPENITVYEGEDVKFTVTAKSDSKTTLDFSDL
WP_047211709.1:423535/1-113    ANENNQQKIELTVSPENITVYEGEDVKFTVTAKSDSKTTLDFSDL
WP_050198886.1:428540/1-113    ANENNQQKIELTVSPENITVYEGEDVKFTVTAKSDSKTTLDFSDL
WP_000477132.1:423535/1-113    ANENNQQKIELTVSPENITVYEGEDVKFTVTAKSDSKTTLDFSDL
WP_000477143.1:428540/1-113    ANENNQQKIELTVSPENITVYEGEDVKFTVTAKSDSKTTLDFSDL
WP_047198467.1:428540/1-113    ANENNQQKIELTVSPENITVYEGEDVKFTVTAKSDSKTTLDFSDL
CAA41384.1:428540/1-113        ANENNQQKIELTVSPENITVYEGEDVKFTVTAKSDSKTTLDFSDL
WP_001880680.1:428540/1-113    ANENNQQKIELTVSPENITVYEGEDVKFTVTAKSDSKTTLDFSDL
WP_000477140.1:428540/1-113    ANENNQQKIELTVSPENITVYEGEDVKFTVTAKSDSKTTLDFSDL
WP_000477139.1:428540/1-113    ANENNQQKIELTVSPENITVYEGEDVKFTVTAKSDSKTTLDFSDL
WP_000477138.1:428540/1-113    ANENNQQKIELTVSPENITVYEGEDVKFTVTAKSDSKTTLDFSDL
WP_070841624.1:428540/1-113    ANENNQQKIELTVSPENITVYEGEDVKFTVTAKSDSKTTLDFSDL
WP_047205529.1:428540/1-113    ANENNQQKIELTVSPENITVYEGEDVKFTVTAKSDSKTTLDFSDL
WP_060800989.1:428540/1-113    ANENNQQKIELTVSPENITVYEGEDVKFTVTAKSDSKTTLDFSDL
WP_000477144.1:428540/1-113    ANENNQQKIELTVSPENITVYEGEDVKFTVTAKSDSKTTLDFSDL
WP_079219395.1:428540/1-113    ANENNQQKIELTVSPENITVYEGEDVKFTVTAKSDSKTTLDFSDL
WP_150411773.1:428540/1-113    ANENNQQKIELTVSPENITVYEGEDVKFTVTAKSDSKTTLDFSDL
WP_001885839.1:428540/1-113    ANENNQQKIELTVSPENITVYEGEDVKFTVTAKSDSKTTLDFSDL
WP_150418747.1:428540/1-113    ANENNQQKIELTVSPENITVYEGEDVKFTVTAKSDSKTTLDFSDL
WP_150415581.1:428540/1-113    ANENNQQKIELTVSPENITVYEGEDVKFTVTAKSDSKTTLDFSDL
AAT10376.1:428540/1-113        ANENNQQKIELTVSPENITVYEGEDVKFTVTAKSDSKTALDFSDL
```

	50 60 70 80
EPU95478.1:428540/1-113	LTKYNPSVSDRISTNYKTNTDNHKIAEITIKNLKLNESQTVTLKA
WP_000295505.1:94206/1-113	LTKYNPSVSDRISTNYKTNTDNHKIAEITIKNLKLNESQTVTLKA
KXA56917.1:93205/1-113	LTKYNPSVSDRISTNYKTNTDNHKIAEITIKNLKLNESQTVTLKA
WP_000477137.1:428540/1-113	LTKYNPSVSDRISTNYKTNTDNHKIAEITIKNLKLNESQTVTLKA
WP_017650912.1:428540/1-113	LTKYNPSVSDRISTNYKTNTDNHKIAEITIKNLKLNESQTVTLKA
WP_132935284.1:428540/1-113	LTKYNPSVSDRISTNYKTNTDNHKIAEITIKNLKLNESQTVTLKA
WP_001933343.1:428540/1-113	LTKYNPSVSDRISTNYKTNTDNHKIAEITIKNLKLNESQTVTLKA
WP_025195781.1:428540/1-113	LTKYNPSVSDRISTNYKTNTDNHKIAEITIKNLKLNESQTVTLKA
WP_132868240.1:428540/1-113	LTKYNPSVSDRISTNYKTNTDNHKIAEITIKNLKLNESQTVTLKA
WP_041980967.1:428540/1-113	LTKYNPSVSDRISTNYKTNTDNHKIAEITIKNLKLNESQTVTLKA
CFQ92576.1:130/1-113	LTKYNPSVSDRISTNYKTNTDNHKIAEITIKNLKLNESQTVTLKA
EPV41091.1:160272/1-113	LTKYNPSVSDRISTNYKTNTDNHKIAEITIKNLKLNESQTVTLKA
WP_049458979.1:428540/1-113	LTKYNPSVSDRISTNYKTNTDNHKIAEITIKNLKLNESQTVTLKA
SQA16054.1:253365/1-113	LTKYNPSVSDRISTNYKTNTDNHKIAEITIKNLKLNESQTVTLKA
BAE45252.1:428540/1-113	LTKYNPSVSDRISTNYKTNTDNHKIAEITIKNLKLNESQTVTLKA
WP_055345127.1:428540/1-113	LTKYNPSVSDRISTNYKTNTDNHKIAEITIKNLKLNESQTVTLKA
WP_017650863.1:428540/1-113	LTKYNPSVSDRISTNYKTNTDNHKIAEITIKNLKLNESQTVTLKA
CNE84135.1:307419/1-113	LTKYNPSVSDRISTNYKTNTDNHKIAEITIKNLKLNESQTVTLKA
WP_060457561.1:428540/1-113	LTKYNPSVSDRISTNYKTNTDNHKIAEITIKNLKLNESQTVTLKA
WP_060458554.1:428540/1-113	LTKYNPSVSDRISTNYKTNTDNHKIAEITIKNLKLNESQTVTLKA
WP_000477135.1:428540/1-113	LTKYNPSVSDRISTNYKTNTDNHKIAEITIKNLKLNESQTVTLKA
WP_121070430.1:428540/1-113	LTKYNPSVSDRISTNYKTNTDNHKIAEITIKNLKLNESQTVTLKA
P27951.1:428540/1-113	LTKYNPSVSDRISTNYKTNTDNHKIAEITIKNLKLNESQTVTLKA
WP_047199360.1:428540/1-113	LTKYNPSVSDRISTNYKTNTDNHKIAEITIKNLKLNESQTVTLKA
WP_017647376.1:428540/1-113	LTKYNPSVSDRISTNYKTNTDNHKIAEITIKNLKLNESQTVTLKA
WP_141445744.1:401513/1-113	LTKYNPSVSDRISTNYKTNTDNHKIAEITIKNLKLNESQTVTLKA
WP_050152779.1:428540/1-113	LTKYNPSVSDRISTNYKTNTDNHKIAEITIKNLKLNESQTVTLKA
WP_000477136.1:428540/1-113	LTKYNPSVSDRISTNYKTNTDNHKIAEITIKNLKLNESQTVTLKA
EPV99179.1:351463/1-113	LTKYNPSVSDRISTNYKTNTDNHKIAEITIKNLKLNESQTVTLKA
WP_071150949.1:419531/1-113	LTKYNPSVSDRISTNYKTNTDNHKIAEITIKNLKLNESQTVTLKA
WP_000477141.1:428540/1-113	LTKYNPSVSDRISTNYKTNTDNHKIAEITIKNLKLNESQTVTLKA
WP_157140373.1:377489/1-113	LTKYNPSVSDRISTNYKTNTDNHKIAEITIKNLKLNESQTVTLKA
WP_000477134.1:428540/1-113	LTKYNPSVSDRISTNYKTNTDNHKIAEITIKNLKLNESQTVTLKA
WP_094754263.1:428540/1-113	LTKYNPSVSDRISTNYKTNTDNHKIAEITIKNLKLNESQTVTLKA
WP_001905993.1:428540/1-113	LTKYNPSVSDRISTNYKTNTDNHKIAEITIKNLKLNESQTVTLKA
WP_017645156.1:423535/1-113	LTKYNPSVSDRISTNYKTNTDNHKIAEITIKNLKLNESQTVTLKA
WP_094969800.1:428540/1-113	LTKYNPSVSDRISTNYKTNTDNHKIAEITIKNLKLNESQTVTLKA
WP_047211709.1:423535/1-113	LTKYNPSVSDRISTNYKTNTDNHKIAEITIKNLKLNESQTVTLKA
WP_050198886.1:428540/1-113	LTKYNPSVSDRISTNYKTNTDNHKIAEITIKNLKLNESQTVTLKA
WP_000477132.1:423535/1-113	LTKYNPSVSDRISTNYKTNTDNHKIAEITIKNLKLNESQTVTLKA
WP_000477143.1:428540/1-113	LTKYNPSVSDRISTNYKTNTDNHKIAEITIKNLKLNESQTVTLKA
WP_047198467.1:428540/1-113	LTKYNPSVSDRISTNYKTNTDNHKIAEITIKNLKLNESQTVTLKA
CAA41384.1:428540/1-113	LTKYNPSVSDRISTNYKTNTDNHKIAEITIKNLKLNESQTVTLKA
WP_001880680.1:428540/1-113	LTKYNPSVSDRISTNYKTNTDNHKIAEITIKNLKLNESQTVTLKA
WP_000477140.1:428540/1-113	LTKYNPSVSDRISTNYKTNTDNHKIAEITIKNLKLNESQTVTLKA
WP_000477139.1:428540/1-113	LTKYNPSVSDRISTNYKTNTDNHKIAEITIKNLKLNESQTVTLKA
WP_000477138.1:428540/1-113	LTKYNPSVSDRISTNYKTNTDNHKIAEITIKNLKLNESQTVTLKA
WP_070841624.1:428540/1-113	LTKYNPSVSDRISTNYKTNTDNHKIAEITIKNLKLNESQTVTLKA
WP_047205529.1:428540/1-113	LTKYNPSVSDRISTNYKTNTDNHKIAEITIKNLKLNESQTVTLKA
WP_060800989.1:428540/1-113	LTKYNPSVSDRISTNYKTNTDNHKIAEITIKNLKLNESQTVTLKA
WP_000477144.1:428540/1-113	LTKYNPSVSDRISTNYKTNTDNHKIAEITIKNLKLNESQTVTLKA
WP_079219395.1:428540/1-113	LTKYNPSVSDRISTNYKTNTDNHKIAEITIKNLKLNESQTVTLKA
WP_150411773.1:428540/1-113	LTKYNPSVSDRISTNYKTNTDNHKIAEITIKNLKLNESQTVTLKA
WP_001885839.1:428540/1-113	LTKYNPSVSDRISTNYKTNTDNHKIAEITIKNLKLNESQTVTLKA
WP_150418747.1:428540/1-113	LTKYNPSVSDRISTNYKTNTDNHKIAEITIKNLKLNESQTVTLKA
WP_150415581.1:428540/1-113	LTKYNPSVSDRISTNYKTNTDNHKIAEITIKNLKLNESQTVTLKA
AAT10376.1:428540/1-113	LTKYNPSVSDRISTNYKTNTDNHKIAEITIKNLKLNESQTVTLKA

```
                                      100       110
EPU95478.1:428540/1-113          KDDSGNVVEKTFTITVQKKEEKQ
WP_000295505.1:94206/1-113       KDDSGNVVEKTFTITVQKKEEKQ
KXA56917.1:93205/1-113           KDDSGNVVEKTFTITVQKKEEKQ
WP_000477137.1:428540/1-113      KDDSGNVVEKTFTITVQKKEEKQ
WP_017650912.1:428540/1-113      KDDSGNVVEKTFTITVQKKEEKQ
WP_132935284.1:428540/1-113      KDDSGNVVEKTFTITVQKKEEKQ
WP_001933343.1:428540/1-113      KDDSGNVVEKTFTITVQKKEEKQ
WP_025195781.1:428540/1-113      KDDSGNVVEKTFTITVQKKEEKQ
WP_132868240.1:428540/1-113      KDDSGNVVEKTFTITVQKKEEKQ
WP_041980967.1:428540/1-113      KDDSGNVVEKTFTITVQKKEEKQ
CFQ92576.1:130/1-113             KDDSGNVVEKTFTITVQKKEEKQ
EPV41091.1:160272/1-113          KDDSGNVVEKTFTITVQKKEEKQ
WP_049458979.1:428540/1-113      KDDSGNVVEKTFTITVQKKEEKQ
SQA16054.1:253365/1-113          KDDSGNVVEKTFTITVQKKEEKQ
BAE45252.1:428540/1-113          KDDSGNVVEKTFTITVQKKEEKQ
WP_055345127.1:428540/1-113      KDDSGNVVEKTFTITVQKKEEKQ
WP_017650863.1:428540/1-113      KDDSGNVVEKTFTITVQKKEEKQ
CNE84135.1:307419/1-113          KDDSGNVVEKTFTITVQKKEEKQ
WP_060457561.1:428540/1-113      KDDSGNVVEKTFTITVQKKEEKQ
WP_060458554.1:428540/1-113      KDDSGNVVEKTFTITVQKKEEKQ
WP_000477135.1:428540/1-113      KDDSGNVVEKTFTITVQKKEEKQ
WP_121070430.1:428540/1-113      KDDSGNVVEKTFTITVQKKEEKQ
P27951.1:428540/1-113            KDDSGNVVEKTFTITVQKKEEKQ
WP_047199360.1:428540/1-113      KDDSGNVVEKTFTITVQKKEEKQ
WP_017647376.1:428540/1-113      KDDSGNVVEKTFTITVQKKEEKQ
WP_141445744.1:401513/1-113      KDDSGNVVEKTFTITVQKKEEKQ
WP_050152779.1:428540/1-113      KDDSGNVVEKTFTITVQKKEEKQ
WP_000477136.1:428540/1-113      KDDSGNVVEKTFTITVQKKEEKQ
EPV99179.1:351463/1-113          KDDSGNVVEKTFTITVQKKEEKQ
WP_071150949.1:419531/1-113      KDDSGNVVEKTFTITVQKKEEKQ
WP_000477141.1:428540/1-113      KDDSGNVVEKTFTITVQKKEEKQ
WP_157140373.1:377489/1-113      KDDSGNVVEKTFTITVQKKEEKQ
WP_000477134.1:428540/1-113      KDDSGNVVEKTFTITVQKKEEKQ
WP_094754263.1:428540/1-113      KDDSGNVVEKTFTITVQKKEEKQ
WP_001905993.1:428540/1-113      KDDSGNVVEKTFTITVQKKEEKQ
WP_017645156.1:423535/1-113      KDDSGNVVEKTFTITVQKKEEKQ
WP_094969800.1:428540/1-113      KDDSGNVVEKTFTITVQKKEEKQ
WP_047211709.1:423535/1-113      KDDSGNVVEKTFTITVQKKEEKQ
WP_050198886.1:428540/1-113      KDDSGNVVEKTFTITVQKKEEKQ
WP_000477132.1:423535/1-113      KDDSGNVVEKTFTITVQKKEEKQ
WP_000477143.1:428540/1-113      KDDSGNVVEKTFTITVQKKEEKQ
WP_047198467.1:428540/1-113      KDDSGNVVEKTFTITVQKKEEKQ
CAA41384.1:428540/1-113          KDDSGNVVEKTFTITVQKKEEKQ
WP_001880680.1:428540/1-113      KDDSGNVVEKTFTITVQKKEEKQ
WP_000477140.1:428540/1-113      KDDSGNVVEKTFTITVQKKEEKQ
WP_000477139.1:428540/1-113      KDDSGNVVEKTFTITVQKKEEKQ
WP_000477138.1:428540/1-113      KDDSGNVVEKTFTITVQKKEEKQ
WP_070841624.1:428540/1-113      KDDSGNVVEKTFTITVQKKEEKQ
WP_047205529.1:428540/1-113      KDDSGNVVEKTFTITVQKKEEKQ
WP_060800989.1:428540/1-113      KDDSGNVVEKTFTITVQKKEEKQ
WP_000477144.1:428540/1-113      KDDSGNVVEKTFTITVQKKEEKQ
WP_079219395.1:428540/1-113      KDDSGNVVEKTFTITVQKKEEKQ
WP_150411773.1:428540/1-113      KDDSGNVVEKTFTITVQKKEEKQ
WP_001885839.1:428540/1-113      KDDSGNVVEKTFTITVQKKEEKQ
WP_150418747.1:428540/1-113      KDDSGNVVEKTFTITVQKKEEKQ
WP_150415581.1:428540/1-113      KDDSGNVVEKTFTITVQKKEEKQ
AAT10376.1:428540/1-113          KDDSGNVVEKTFTITVQKKEEKQ
```

Appendix Figure S4: Alignment of IgI3 domain sequences from β protein of GBS. Alignment of IgI3 domain sequences of β protein in the BLAST database.

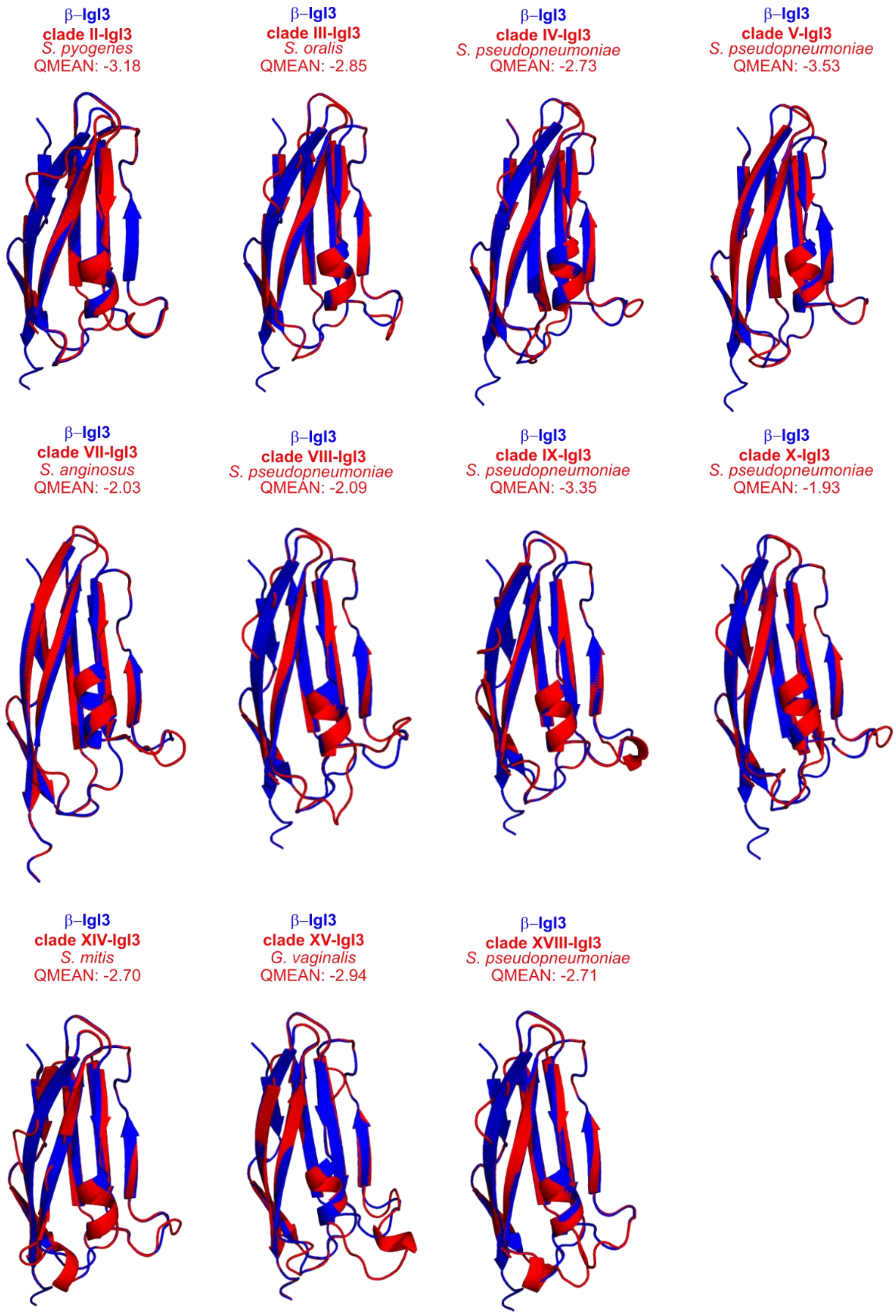

Appendix Figure S5: Predicted structures of IgI3 homologs. Structure of β-IgI3 homologs was predicted using SWISS-MODEL in which the β-IgI3 was used as a template. The predicted structures of 11 sequences (red) are superimposed onto β-IgI3 structure (blue), with the clade, bacterial species and QMEAN score of the model shown.

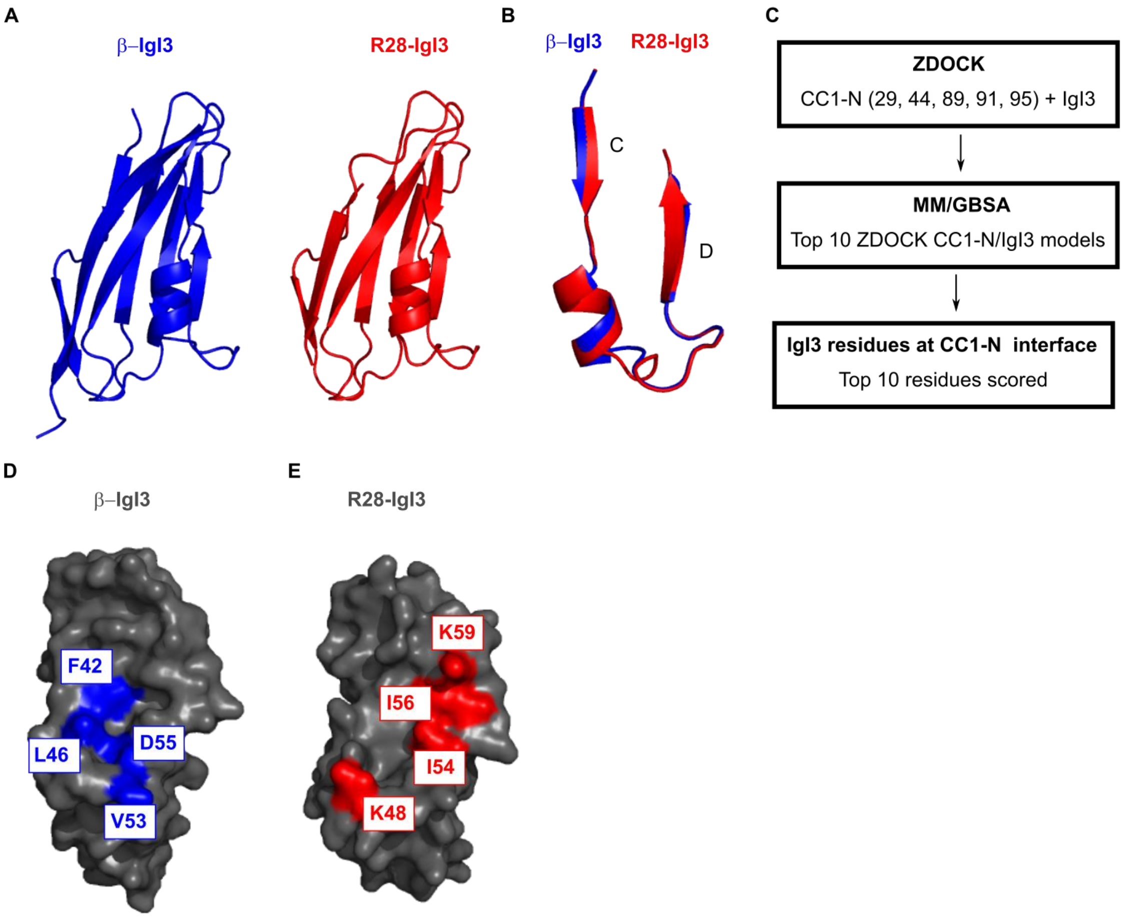

Appendix Figure S6: IgI3 domains bind CEACAM1 through alternative binding pockets.

A) Structure of β-IgI3 and predicted structure of R28-IgI3. **B)** Superposition of the known IgI3 domain from β protein (blue) onto predicted structure of IgI3 domain from R28 (red), only the *C* to *D* strand region is shown. **C)** Pipeline showing prediction of key and unfavourable IgI3 residues at the CEACAM1 binding interface. β-IgI3 and R28-IgI3 docking to CEACAM1-N (CC1-N) was simulated 50 times using ZDOCK. CC1-N residues 29, 44, 89, 91 and 95 were set as important binding residues. Each simulation was analysed by MM/GBSA analysis to quantify the free energy binding for each residue independently. **D)** Surface structure of β-IgI3 shown, with key (F42, L46 and V53) or unfavourable (D55) residues required for CC1-N binding shown in blue. **E)** Surface structure of R28-IgI3 shown, with key (K48, I54, I56 and K59) residues required for CC1-N binding shown in red.

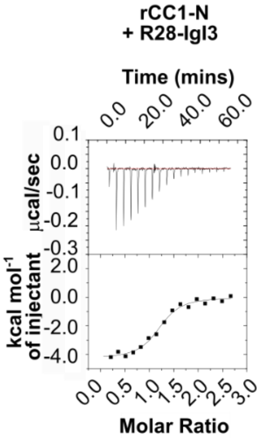

Appendix Figure S7: Isothermal titration calorimetry (ITC) binding curve of CEACAM1-N and R28-IgI3. Representative ITC plot for rCEACAM1 (CC1)-N domain and R28-IgI3 duplicates. Experiments were performed using an iTC200 instrument (GE Healthcare), at 25 °C with 16 injections of 2.42 µL aliquots. All data were analyzed using Origin 7.0 software.

Species	Strain	Notes	Growth*	Reference[$]
Streptococcus pyogenes (GAS)	M1 5448		TH-Y, 37ºC	[1]
	M2		TH-Y, 37ºC	N. van Sorge, this study
	M3		TH-Y, 37ºC	N. van Sorge, this study
	M6		TH-Y, 37ºC	N. van Sorge, this study
	M11		TH-Y, 37ºC	N. van Sorge, this study
	M12		TH-Y, 37ºC	N. van Sorge, this study
	M89		TH-Y, 37ºC	N. van Sorge, this study
	AL368		TH-Y, 37ºC	[2]
Streptococcus agalactiae (GBS)	A909	Serotype Ia; *bac* positive	TH, 37ºC	[3]
	A909Δ*bac*		TH, 37ºC	[4]
	A909Δ*bac + pLZ.bac*		TH + k + s, 37ºC	[4]
	A909Δ*bca*		TH + k, 37ºC	E. Movert, T. Areschoug, Lund University
	A909Δ*cap*		TH + k, 37ºC	E. Movert, T. Areschoug, Lund University
	A909Δ*bac*Δ*cap*		TH + k, 37ºC	E. Movert, T. Areschoug, Lund University
	BS39	Serotype Ia; *bac* negative; invasive isolate	TH, 37ºC	G. Lindahl, this study
	515	Serotype Ia; *bac* negative	TH, 37ºC	[5]
	BS22	Serotype Ib; *bac* positive	TH, 37ºC	[5]
	H36B	Serotype Ib; *bac* positive	TH, 37ºC	ATCC #BAA-1174
	BS29	Serotype II; *bac* positive; invasive isolate	TH, 37ºC	G. Lindahl, this study
	18RS21	Serotype II; *bac* negative	TH, 37ºC	ATCC #BAA1175
	BM110	Serotype III; *bac* negative	TH, 37ºC	[6]
	COH1	Serotype III; *bac* negative	TH, 37ºC	ATCC #BAA-1176
	SBL3066	Serotype V; *bac* positive; invasive isolate	TH, 37ºC	G. Lindahl, this study
	NCTC10/84	Serotype V; *bac* negative	TH, 37ºC	ATCC #49447
	SB35	*bac* positive	TH, 37ºC	[6]
	SB10	*bac* positive	TH, 37ºC	[5]
	SB20	Serotype III; *bac* positive	TH, 37ºC	[5]
	BS26	*bac* positive	TH, 37ºC	[5]
	H1147 (PHEGBS0159)	*alp3* positive	TH, 37ºC	[7]
Streptococcus zooepidemicus (GCS)	CS2	Invasive infection	TH, 37ºC	G. Lindahl, this study
	CS3	Invasive infection	TH, 37ºC	G. Lindahl, this study
	CS4	Invasive infection	TH, 37ºC	G. Lindahl, this study

	CS7	Invasive infection	TH, 37ºC	G. Lindahl, this study
	CS8	Invasive infection	TH, 37ºC	G. Lindahl, this study
	L31	Invasive infection	TH, 37ºC	G. Lindahl, this study
	L32	Invasive infection	TH, 37ºC	G. Lindahl, this study
Streptococcus dysgalactiae (GGS)	G148		TH, 37ºC	[8]
	GS1	Invasive infection	TH, 37ºC	G. Lindahl, this study
	GS2	Invasive infection	TH, 37ºC	G. Lindahl, this study
	GS3	Invasive infection	TH, 37ºC	G. Lindahl, this study
	GS4	Invasive infection	TH, 37ºC	G. Lindahl, this study
	GS5	Invasive infection	TH, 37ºC	G. Lindahl, this study
	GS6	Invasive infection	TH, 37ºC	G. Lindahl, this study
	GS7	Invasive infection	TH, 37ºC	G. Lindahl, this study
	GS8	Invasive infection	TH, 37ºC	G. Lindahl, this study
	GS9	Invasive infection	TH, 37ºC	G. Lindahl, this study
	L33	Invasive infection	TH, 37ºC	G. Lindahl, this study
	L34	Invasive infection	TH, 37ºC	G. Lindahl, this study
Streptococcus pneumoniae	PBCN22		TH, 37ºC	UMC Utrecht, this study
	D39		TH, 37ºC	NCTC #7466
	TIGR4		TH, 37ºC	BAA-334
	PBCN57		TH, 37ºC	UMC Utrecht, this study
	PBCN79		TH, 37ºC	UMC Utrecht, this study
	PBCN24		TH, 37ºC	UMC Utrecht, this study
	PBCN133		TH, 37ºC	UMC Utrecht, this study
Enterococcus faecium	E8284		TH, 37ºC	[9]
	E4413		TH, 37ºC	[9]
	E656		TH, 37ºC	[9]
	E4227		TH, 37ºC	[9]
	E7313		TH, 37ºC	[9]
	E7098		TH, 37ºC	[9]
Enterococcus faecalis	E02500	Human blood isolate	TH, 37ºC	UMC Utrecht, this study
	E02504	Human blood isolate	TH, 37ºC	UMC Utrecht, this study
	E02608	Human blood isolate	TH, 37ºC	UMC Utrecht, this study
	E02835	Human blood isolate	TH, 37ºC	UMC Utrecht, this study

	E4877	Human faecal isolate	TH, 37ºC	UMC Utrecht, this study
	E6568	Human faecal isolate	TH, 37ºC	UMC Utrecht, this study
Staphylococcus aureus	MW2		TSB, 37ºC	ATCC #BAA-1707
	MRSA252		TSB, 37ºC	ATCC #BAA-1720
	PS66		TSB, 37ºC	U. Bläsi, Vienna
	80286		TSB, 37ºC	[10]
	USA300		TSB, 37ºC	ATCC #BAA-1556
	N315		TSB, 37ºC	[11]
	Newman		TSB, 37ºC	[12]

Appendix Table S1. Bacterial strains used in this study. * Growth media as follows, Todd-Hewitt (TH) broth, Todd-Hewitt + 0.6% yeast (TH-Y) broth, Tryptic Soy (TS) broth. Media was supplemented with 500 µg/ml kanamycin (k) and/or 70 µg/ml spectinomycin (s). [$] Strain names available from American Type Culture Collection (ATCC) or National Collection of Type Cultures (NCTC). [1] Chatellier, S. *et al.* Genetic relatedness and superantigen expression in group A Streptococcus serotype M1 isolates from patients with severe and nonsevere invasive diseases. *Infect Immun* **68**, 3523–3534 (2000). [2] Stålhammar-Carlemalm, M., Areschoug, T., Larsson, C. & Lindahl, G. The R28 protein of Streptococcus pyogenes is related to several group B streptococcal surface proteins, confers protective immunity and promotes binding to human epithelial cells. *Mol Microbiol* **33**, 208–219 (1999). [3] Michel, J. L. *et al.* Large, identical, tandem repeating units in the C protein alpha antigen gene, bca, of group B streptococci. *Proc Natl Acad Sci U S A* **89**, 10060–10064 (1992). [4] Areschoug, T., Stålhammar-Carlemalm, M., Karlsson, I. & Lindahl, G. Streptococcal β protein has separate binding sites for human factor H and IgA-Fc. *J Biol Chem* **277**, 12642–12648 (2002). [5] Areschoug, T., Linse, S., Stålhammar-Carlemalm, M., Hedén, L. O. & Lindahl, G. A proline-rich region with a highly periodic sequence in streptococcal β protein adopts the polyproline II structure and is exposed on the bacterial surface. *J Bacteriol* **184**, 6376–6383 (2002). [6] Stålhammar-Carlemalm, M., Stenberg, L. & Lindahl, G. Protein Rib: a novel group B streptococcal cell surface protein that confers protective immunity and is expressed by most strains causing invasive infections. *J Exp Med* **177**, 1593–603 (1993). [7] Jauneikaite, E. *et al.* Serial clustering of late-onset group B streptococcal infections in the neonatal unit: a genomic re-evaluation of causality. *Clin Infect Dis* **67**, 854–860 (2018). [8] Kronvall, G., Simmons, A., Myhre, E. B. & Jonsson, S. Specific absorption of human serum albumin, immunoglobulin A, and immunoglobulin G with selected strains of group A-and G streptococci. *Infect Immun* **25**, 1–10 (1979). [9] Arredondo-Alonso, S. *et al.* Plasmids shaped the recent emergence of the major nosocomial pathogen Enterococcus faecium. *mBio* **11**, e03284-19 (2020). [10] Winstel, V. *et al.* Wall teichoic acid glycosylation governs Staphylococcus aureus nasal colonization. *mBio* **6**,

e00632-15 (2015). [11] Kuroda, M. *et al.* Whole genome sequencing of meticillin-resistant Staphylococcus aureus. *Lancet* **357**, 1225–40 (2001). [12] Baba, T., Bae, T., Schneewind, O., Takeuchi, F. & Hiramatsu, K. Genome sequence of Staphylococcus aureus strain newman and comparative analysis of staphylococcal genomes: Polymorphism and evolution of two major pathogenicity islands. *J Bacteriol* **190**, 300–310 (2008).

Vector	Protein	Expression system
pcDNA3.4.CEACAM1	rCEACAM1-His	Expi293F
pcDNA3.4.CEACAM3	rCEACAM3-His	Expi293F
pcDNA3.4.CEACAM5	rCEACAM5-His	Expi293F
pcDNA3.4.CEACAM6	rCEACAM6-His	Expi293F
pcDNA3.4.CEACAM8	rCEACAM8-His	Expi293F
pRSET-C-CEACAM1N	rCEACAM1-N-His	*E. coli* RG
pRSET-C-CEACAM1NΔF29A	rCEACAM1-NΔF29A-His	*E. coli* RG
pRSET-C-CEACAM1NΔQ44A	rCEACAM1-NΔQ44A-His	*E. coli* RG
pRSET-C-CEACAM1NΔA49V	rCEACAM1-NΔA49V-His	*E. coli* RG
pRSET-C-CEACAM1NΔQ89A	rCEACAM1-NΔQ89A-His	*E. coli* RG
pRSET-C-CEACAM1NΔL95A	rCEACAM1-NΔL95A-His	*E. coli* RG
pRSET-C-CEACAM1NΔV96A	rCEACAM1-NΔV96A-His	*E. coli* RG
pRSET-C-CEACAM1NΔN97A	rCEACAM1-NΔN97A-His	*E. coli* RG
pRSET-C-CEACAM1A1B1A2	rCEACAM1-A1B1A2-His	*E. coli* RG
pRSET-C-CEACAM3N	rCEACAM3-N-His	*E. coli* RG
pRSET-C-CEACAM5N	rCEACAM5-N-His	*E. coli* RG
pRSET-C-CEACAM6N	rCEACAM6-N-His	*E. coli* RG
pRSET-C-CEACAM8N	rCEACAM8-N-His	*E. coli* RG
pET21d-CEACAM1N	rCEACAM1-N	*E. coli* RG
pET21d-CEACAM1NΔF29A	rCEACAM1-NΔF29A	*E. coli* RG
pET21d-CEACAM1NΔQ44A	rCEACAM1-NΔQ44A	*E. coli* RG
pET21d-CEACAM1NΔA49V	rCEACAM1-NΔA49V	*E. coli* RG
pET21d-CEACAM1NΔQ89A	rCEACAM1-NΔQ89A	*E. coli* RG
pET21d-CEACAM1NΔL95A	rCEACAM1-NΔL95A	*E. coli* RG
pET21d-CEACAM1NΔV96A	rCEACAM1-NΔV96A	*E. coli* RG
pET21d-CEACAM1NΔN97A	rCEACAM1-NΔN97A	*E. coli* RG
pRSET-C-B6N	rB6N-His	*E. coli* RG
pRSET-C-IgABR	rIgABR-His	*E. coli* RG
pRSET-C-B6C	rB6C-His	*E. coli* RG
pRSET-C-β-IgSF / β-IgI3	rβIgSF-His, β-IgI3-His	*E. coli* RG
	rβIgI3ΔF42A-His	*E. coli* RG
	rβIgI3ΔL45A-His	*E. coli* RG
	rβIgI3ΔL46A-His	*E. coli* RG
	rβIgI3ΔS52A-His	*E. coli* RG
	rβIgI3ΔV53A-His	*E. coli* RG
	rβIgI3ΔD55A-His	*E. coli* RG
pRSET-C-β75KN	rβ75KN-His	*E. coli* RG
pRSET-C-R28-IgI3	rR28-IgI3-His	*E. coli* RG

Appendix Table S2: Expression vectors constructs. Open reading frames (ORFs) coding the extracellular domains of CEACAMs were cloned into pcDNA3.4 vectors, and proteins were expressed in Expi293F cells. ORFs coding the N domains of CEACAMs, the A1B1A2 domain of CEACAM1 and β protein domains were cloned into pRSET-C vectors, and proteins were expressed in *E. coli*.

	K_D (nM)	ΔH (kcal mol^{-1})	TΔS (kcal mol^{-1})
**β-IgSF**			
rCC1-N	96±2	-4.7±0.3	+4.9
rCC3-N	No Binding Observed		
rCC5-N	152±27	-2.2±0.1	+7.1
rCC6-N	No Binding Observed		
rCC8-N	No Binding Observed		

Appendix Table S3: Isothermal Titration Calorimetry (ITC) binding curves constants and thermodynamic paramters for CEACAM-N and β-IgSF interactions. Experiments were performed using β-IgI3 and N domains of (r)CEACAM1 (CC1), CEACAM3 (CC3), CEACAM5 (CC5), CEACAM6 (CC6) and CEACAM8 (CC8) on an iTC200 instrument (GE Healthcare), at 25 °C with 16 injections of 2.42 µL aliquots. All data were analyzed using Origin 7.0 software.

	**β-IgI-CEACAM1-N**
**Data collection**	
Space group	I4122
Cell dimensions	
a, *b*, *c* (Å)	131.62 131.62 257.07
α, β, γ (°)	90.00 90.00 90.00
Resolution (Å)	48.57-3.25
R_{merge}	0.076 (1.386)
$R_{p.i.m}$	0.035 (0.635)
$I / \sigma I$	18.2 (2.2)
Completeness (%)	99.8 (99.8)
Redundancy	10.7 (10.9)
$CC_{1/2}$	0.999 (0.919)
**Refinement**	
Resolution (Å)	48.57-3.25
No. reflections	18198
R_{work} / R_{free}	21.8/24.4
No. atoms	
Protein	3311
Water	1
Ligand	37
B-factors	
Protein	161.19
Water	152.40
Ligands	193.10
R.m.s. deviations	
Bond lengths (Å)	0.004
Bond angles (°)	1.422

Appendix Table S4. **Data collection and refinement statistics for β-IgI3 and CEACAM1 (CC1)-N**. Values in parentheses are for highest-resolution shell.

β-IgI3 atom	Distance (Å)	CC1-N atom
L40	3.88	S93 [C]
	3.86	S93 [O]
	3.65	D94 [N]
	3.24	D94 [CA]
	3.27	D94 [CB]
D41 [CA]	3.53	S93 [O]
D41 [OD1]	3.66	S93 [CB]
D41 [C]	3.49	S93 [O]
F42 [N]	3.72	S93 [C]
	2.57	S93 [O]
F42 [CA]	3.40	S93 [O]
F42 [CB]	3.34	S93 [O]
	3.66	L95 [CD1]
F42 [CG]	3.77	S93 [O]
	3.65	L95 [CD2]
	3.97	L95 [CD1]
F42 [CD2]	3.36	S93 [O]
	3.93	D94 [CA]
	3.59	D94 [C]
	3.75	D94 [O]
	3.89	L95 [N]
	3.57	L95 [CB]
	3.59	L95 [CD2]
	3.84	L95 [CG]
	3.84	L95 [CD1]
F42 [CE2]	3.79	D94 [C]
	3.84	D94 [O]
	3.79	L95 [CB]
	3.94	L95 [CD2]
F42 [C]	3.93	S93 [O]
S43 [OG]	3.95	S93 [OG]
L46 [CB]	3.79	F29 [CG]
	3.54	F29 [CD2]
	3.52	F29 [CE2]
	3.99	F29 [CE1]
	3.75	F29 [CZ]
L46 [CG]	3.68	F29 [CD1]
	3.84	F29 [CD2]
L46 [CD1]	3.34	F29 [CB]
	3.62	F29 [CD1]
	3.39	F29 [CG]
	3.39	F29 [CD2]
L46 [CD2]	3.51	L95 [CD1]
L46 [C]	3.71	F29 [CZ]
L46 [O]	3.79	F29 [CZ]
T47 [N]	3.96	F29 [CE1]
	3.80	F29 [CZ]
T47 [CG2]	3.54	F29 [CE1]
N50 [ND2]	3.36	I91 [CD1]
N50 [O]	3.19	F29 [CE2]
	3.35	F29 [CZ]
P51 [O]	3.67	A49 [CB]
S52 [CA]	3.33	T56 [OG1]

S52 [CB]	2.94	T56 [OG1]
	3.71	T56 [CB]
V53 [CB]	3.89	Y31 [O]
V53 [CG1]	3.97	G30 [C]
	4.00	G30 [O]
	3.81	Y31 [C]
	3.80	Y31 [N]
	3.41	S32 [N]
	3.54	I91 [CG1]
	3.36	I91 [CD1]
	3.93	S32 [CB]
V53 [CG2]	3.69	G30 [C]
	3.76	Y31 [N]
	3.46	G30 [CA]
	3.98	Y48 [N]
	3.54	Y48 [C]
	3.32	Y48 [O]
	3.65	A49 [N]
	3.89	A49 [CA]
V53 [C]	3.91	S32 [OG]
	3.62	Q44 [NE2]
V53 [O]	3.93	S32 [OG]
	4.00	G47 [CA]
	3.66	S32 [CB]
	3.57	Q44 [CD]
	3.88	Q44 [OE1]
	2.50	Q44 [NE2]
S54 [N]	3.95	S32 [OG]
	3.81	I91 [CD1]
S54 [CA]	3.29	Y34 [OH]
S54 [C]	3.47	Y34 [OH]
S54 [O]	3.22	I91 [CD1]
D55 [N]	3.24	Y34 [OH]
D55 [CG]	3.74	G41 [N]
	3.80	G41 [CA]
D55 [OD1]	3.82	G41 [CA]
D55 [OD2]	3.50	V39 [O]
	3.75	D40 [C]
	2.90	G41 [N]
	3.37	G41 [CA]
I57 [CG2]	4.00	L95 [CD2]
T59 [CB]	3.80	L95 [O]
T59 [OG1]	2.86	L95 [O]
T59 [CG2]	3.76	L95 [CB]
	3.45	L95 [CD2]
	3.98	L95 [O]
	3.56	L95 [CG]
Y61 [CZ]	3.45	D94 [O]
Y61 [OH]	3.89	D94 [CB]
	3.77	D94 [C]
	2.66	D94 [O]
Y61 [CE2]	3.43	V96 [CG1]
	3.43	D94 [O]
	3.98	L95 [O]

Y61 [CD2]	3.99	V96 [CG1]

Appendix Table S5. Contact sites in (β-IgI3)(CEACAM1-N) complex. β-IgI3 atoms within 4.0Å of CEACAM1-N (CC1-N) atoms are displayed as calculated using Ncont from the CCP4 suite of programs (Winn *et al*, 2011).

	K_D (nM)	ΔH (kcal mol^{-1})	TΔS (kcal mol^{-1})
**Binding to rCC1-N**			
β-IgI3	96±2	-4.7±0.3	+4.9
β-IgI3^{F42A}	16±15	-6.3±0.2	+5.2
β-IgI3^{L45A}	234±16	-4.7±0.1	+4.3
β-IgI3^{L46A}	No Binding Observed		
β-IgI3^{S52A}	85±20	-4.8±0.1	+4.9
β-IgI3^{V53A}	562±44	-4.3±0.0	+4.3
β-IgI3^{D55A}	690±52	-3.1±0.1	+5.3
**Binding to β-IgI3**			
rCC1-N	96±2	-4.7±0.3	+4.9
rCC1-N^{F29A}	No Binding Observed		
rCC1-N^{Q44A}	996±116	-7.6±0.1	+0.6
rCC1-N^{Q89A}	No Binding Observed		
rCC1-N^{I91A}	No Binding Observed		
rCC1-N^{L95A}	1350±460	-2.3±0.1	+5.7
rCC1-N^{V96A}	370±4	-7.0±0.1	+1.8
rCC1-N^{N97A}	490±120	-9.2±0.4	-0.5

Appendix Table S6: Isothermal Titration Calorimetry (ITC) binding curves constants and thermodynamic paramters for CEACAM1 (CC1)-N and β-IgI3 mutants. Experiments were performed using an iTC200 instrument (GE Healthcare), at 25 °C with 16 injections of 2.42 µL aliquots. All data were analyzed using Origin 7.0 software.

Cell Line	Description
Expi293F	For expression of soluble recombinant proteins
HeLa.EV	HeLa cells carry empty vector
HeLa.CEACAM1	HeLa cells stably expressing CEACAM1
HeLa.CEACAM3	HeLa cells stably expressing CEACAM3
HeLa.CEACAM5	HeLa cells stably expressing CEACAM5
HeLa.CEACAM6	HeLa cells stably expressing CEACAM6
HeLa.CEACAM8	HeLa cells stably expressing CEACAM8
CHO.EV	Chinese Hamster Ovary cells carrying empty vector
CHO.CEACAM1	Chinese Hamster Ovary cells stably expressing CEACAM1
CHO.CEACAM3	Chinese Hamster Ovary cells stably expressing CEACAM3
CHO.CEACAM5	Chinese Hamster Ovary cells stably expressing CEACAM5
CHO.CEACAM6	Chinese Hamster Ovary cells stably expressing CEACAM6
CHO.CEACAM8	Chinese Hamster Ovary cells stably expressing CEACAM8

Appendix Table S7: Cell lines used in this study.

**Target**	**Clone or catalog #**	**Conjugate**	**Manufacturer**	**Link**
N-terminal domain of CEACAM1, CEACAM3 and CEACAM5	CC1/3/5-Sab	-	LeukoCom GmbH, Essen, Germany.	
A1B1 domain of CEACAM1	B3-17	-	LeukoCom GmbH, Essen, Germany.	https://www.kerafast.com/productgroup/860/ceacam1cd66a-antibodies
A1B1 domain of CEACAM1	C5-1X	-	LeukoCom GmbH, Essen, Germany.	https://www.kerafast.com/productgroup/860/ceacam1cd66a-antibodies
A1B1A2B2A3B3 domain of CEACAM5	5C8C4	-	LeukoCom GmbH, Essen, Germany.	https://ximbio.com/reagent/153325/anti-ceacam5-cd66e-5c8c4-monoclonal-antibody
Mouse IgG	#R0480	PE	LeukoCom GmbH, Essen, Germany.	https://www.agilent.com/store/en_US/Prod-R048001-2/R048001-2?navAction=push&catId=SubCat2ECS_146502&pCatName=Secondary%20Antibody%20Conjugates
Mouse IgG	#1036-05	HRP	LeukoCom GmbH, Essen, Germany.	https://www.southernbiotech.com/?catno=1036-05&type=Polyclonal#&panel2-1
Rabbit IgG	#AB_2632461	PE	LeukoCom GmbH, Essen, Germany.	https://www.jacksonimmuno.com/catalog/products/111-117-008/Goat-Rabbit-IgG-Fc-R-Phycoerythrin

6xHis	#AD1.1.10	FITC	LeukoCom GmbH, Essen, Germany.	https://www.thermofisher.com/antibody/product/6x-His-Tag-Antibody-clone-AD1-1-10-Monoclonal/MA1-81891

Appendix Table S8: Antibodies used in this study.
