# Supplementary material for: Bacterial protein domains with a novel Ig‐like fold target human CEACAM receptors
Source: EMBO J. 2021 Feb 1;40(7):e106103. doi: 10.15252/embj.2020106103 (PMC8013792; doi:10.15252/embj.2020106103)
Supplement: Supplementary file 2 — Expanded View Figures PDF [file EMBJ-40-e106103-s002.pdf]

Expanded View Figures

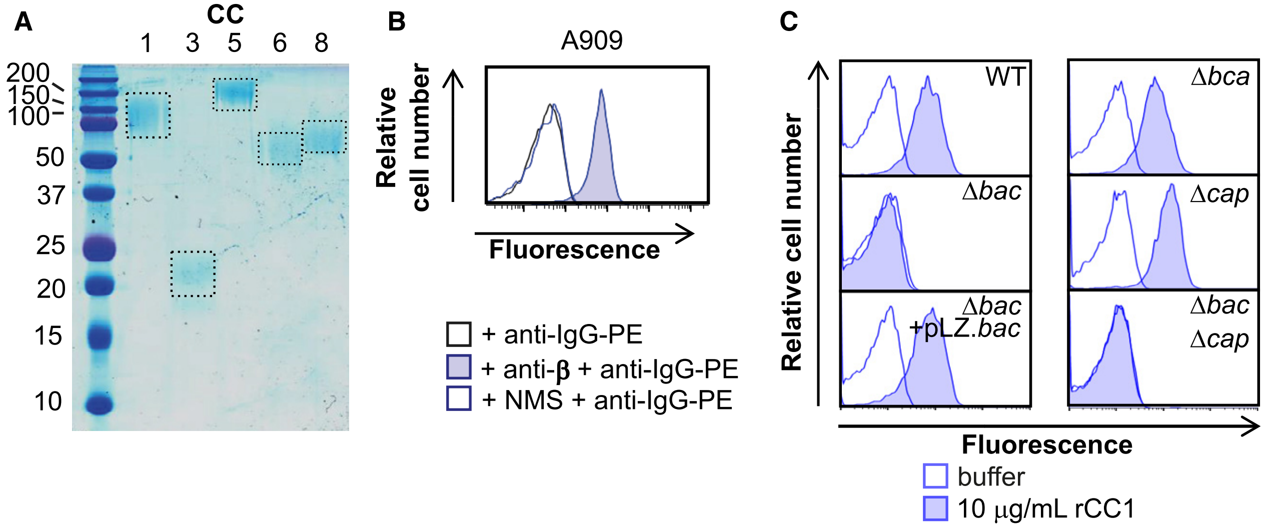

Figure EV1. GBS binds CEACAM1 receptor through β protein.

A Expression and purification of 6xHis tagged recombinant (r)CEACAM1 (CC1), CEACAM3 (CC3), CEACAM5 (CC5), CEACAM6 (CC6) and CEACAM8 (CC8) from a eukaryotic expression system. Dotted boxes signal proteins of expected size.

B Mouse anti-β serum, but not normal mouse serum (NMS), detected expression of β protein in wild-type A909 strain.

C Binding of rCC1 (10 μg/ml) to GBS strain A909 was mediated via the *bac* gene, as demonstrated by comparison of CC1 binding to wild type, Δ*bac* and complemented strains (Δ*bac* + pLZ.*bac*). Mutation of genes encoding other surface proteins (α protein encoded by *bca*) or surface structures (capsule encoded by *cap*) did not impact rCC1 binding. Data representative of $n = 3$ replicates.

Data information: Fluorescence of bacteria in (B and C) was measured by flow cytometry.

© 2021 The Authors

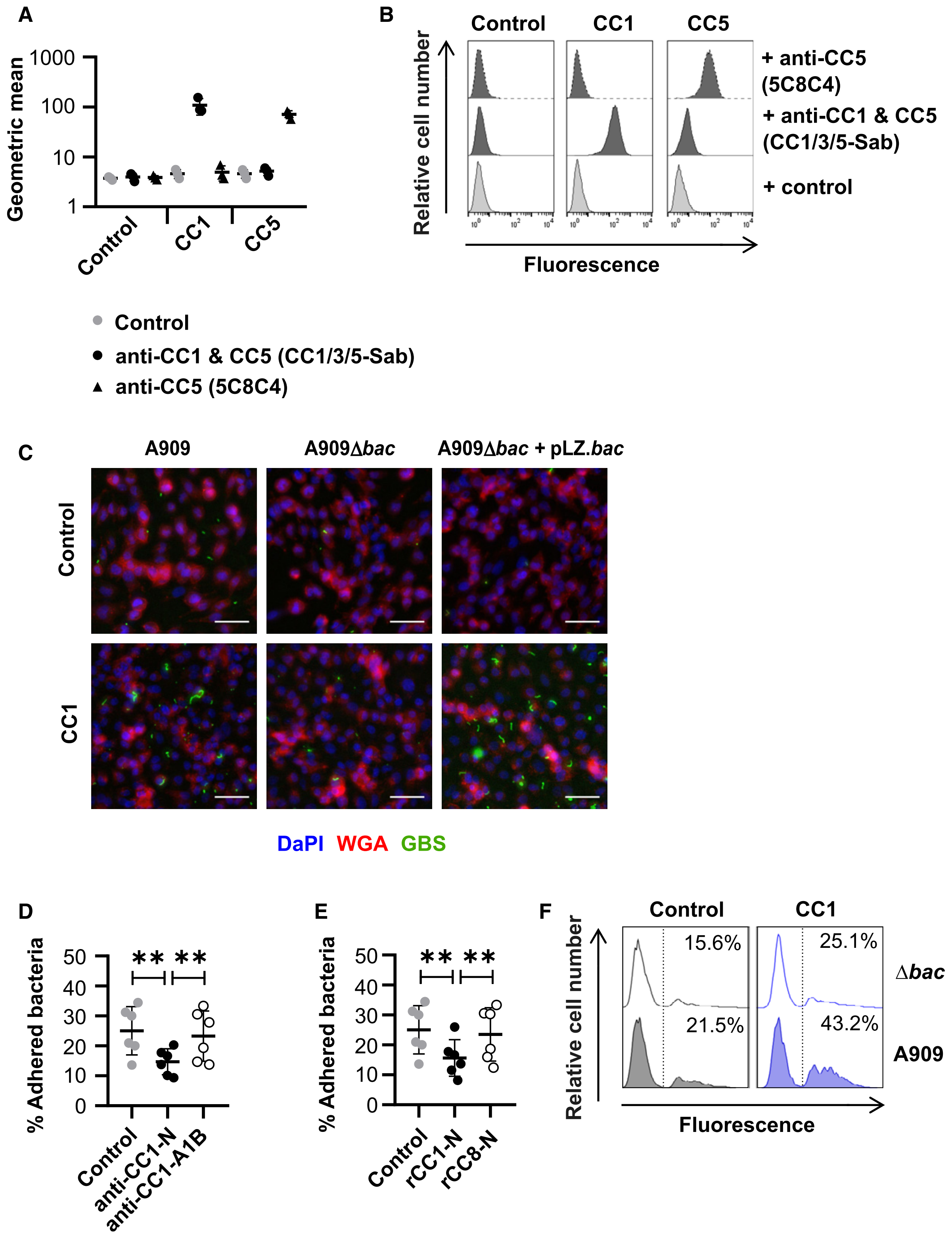

Figure EV2.

© 2021 The Authors

◀ **Figure EV2. GBS exploit CEACAM1 for colonisation of epithelial cells.**

A, B Expression of human CEACAM1 (CC1) and CEACAM5 (CC5) on detached CHO cell lines, as measured by flow cytometry. CC1/3/5-Sab mAb recognises the N-terminal domain of CC1, CC3 and CC5, whilst 5C8C4 mAb recognises CC5. Mean and standard deviation of data from $n = 3$ replicates is shown in (A), and representative flow cytometry plots are show in (B).

C Widefield microscopy imaging of FITC-labelled GBS A909, Δ*bac* or Δ*bac* + pLZ.*bac* strain adhesion to human CC1-expressing or empty vector control CHO transfectants. Cell membranes were stained with AF-647 conjugated wheat germ agglutinin (WGA) (red) and nuclei with DAPI (blue). Scale bars represent 40 μm.

D Adhesion of GBS A909 to CC1 expressing CHO cells was inhibited by pre-incubating cells with anti-CC1-N (clone CC1/3/5-Sab) mAb but not anti-CC1-A1B (clone B3-17) mAb. Data were analysed by repeated one-way ANOVA with Sidak's multiple comparisons. **$P < 0.01$. Mean and SD values are reported for $n = 6$ independent experiments.

E Adhesion of GBS A909 to CC1 expressing CHO cells was inhibited by pre-incubating bacteria with 30 μg/ml rCC1-N but not rCEACAM8 (CC8)-N. Data were analysed by repeated one-way ANOVA with Sidak's multiple comparisons. **$P < 0.01$. Mean and SD values are reported for $n = 6$ independent experiments.

F Adhesion of FITC-labelled A909 strains to human CC1-expressing or control CHO transfectants at an MOI of 10. Cell lines were detached and then incubated with FITC-labelled A909 strains for 30 min at 4°C. Fluorescence of cells was measured by flow cytometry analysis.

Data information: Fluorescence of cells in (A, B and F) was measured by flow cytometry. The number of adhered bacteria to cell monolayers in (D and E) was quantified by growth on Todd-Hewitt agar and enumeration of colony forming units (CFU).

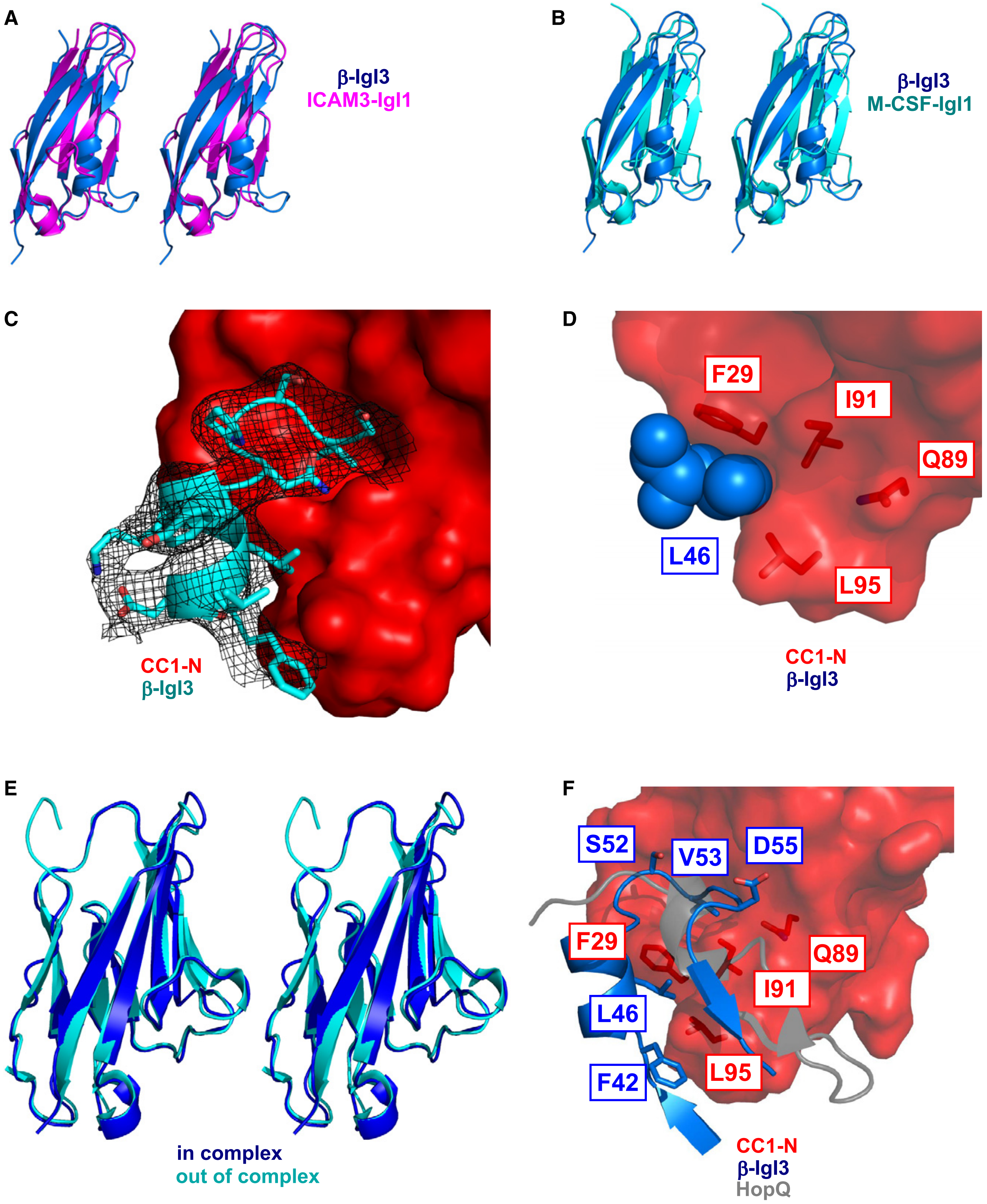

Figure EV3.

 © 2021 The Authors

Figure EV3. Crystal stucture of (β-IgI3)-(CEACAM1) interface.

A Superposition of β-IgI3 (cyan) with that of IgI1 domain of ICAM3, shown in stereo view.
B Superposition of β-IgI3 (cyan) with that of IgI1 domain of M-CSF, shown in stereo view.
C An electron density map showing β-IgI3 bound to CC1-N.
D Focus of the critical β-IgI3 residue L46 and interacting residues in CEACAM1 (CC1)-N. A r.m.s.d. of 0.74 Å is observed.
E Superposition of CC1-N (pdb 2gk2, cyan) with that of CC1-N in complex with β-IgI3 (blue). A r.m.s.d. of 0.74 Å is observed.
F Superposition of the β-IgI3 and CEACAM1 (CC1)-N complex (blue and red, respectively) and HopQ and CC1-N complex (grey and red, respectively).

Figure EV4. Isothermal Titration Calorimetry (ITC) binding curves.

A, B (A) rCEACAM1 (CC1)-N domain and β-IgI3 mutants, and (B) rCC1-N domain mutants and β-IgI3. ITC binding curves for wild-type β-IgI3 (formally called β-IgSF) and wild-type rCC1-N are duplicated from Fig 3A for ease of comparison. Experiments were performed using an iTC200 instrument (GE Healthcare), at 25°C with 16 injections of 2.42 μl aliquots. All data were analysed using Origin 7.0 software.

© 2021 The Authors

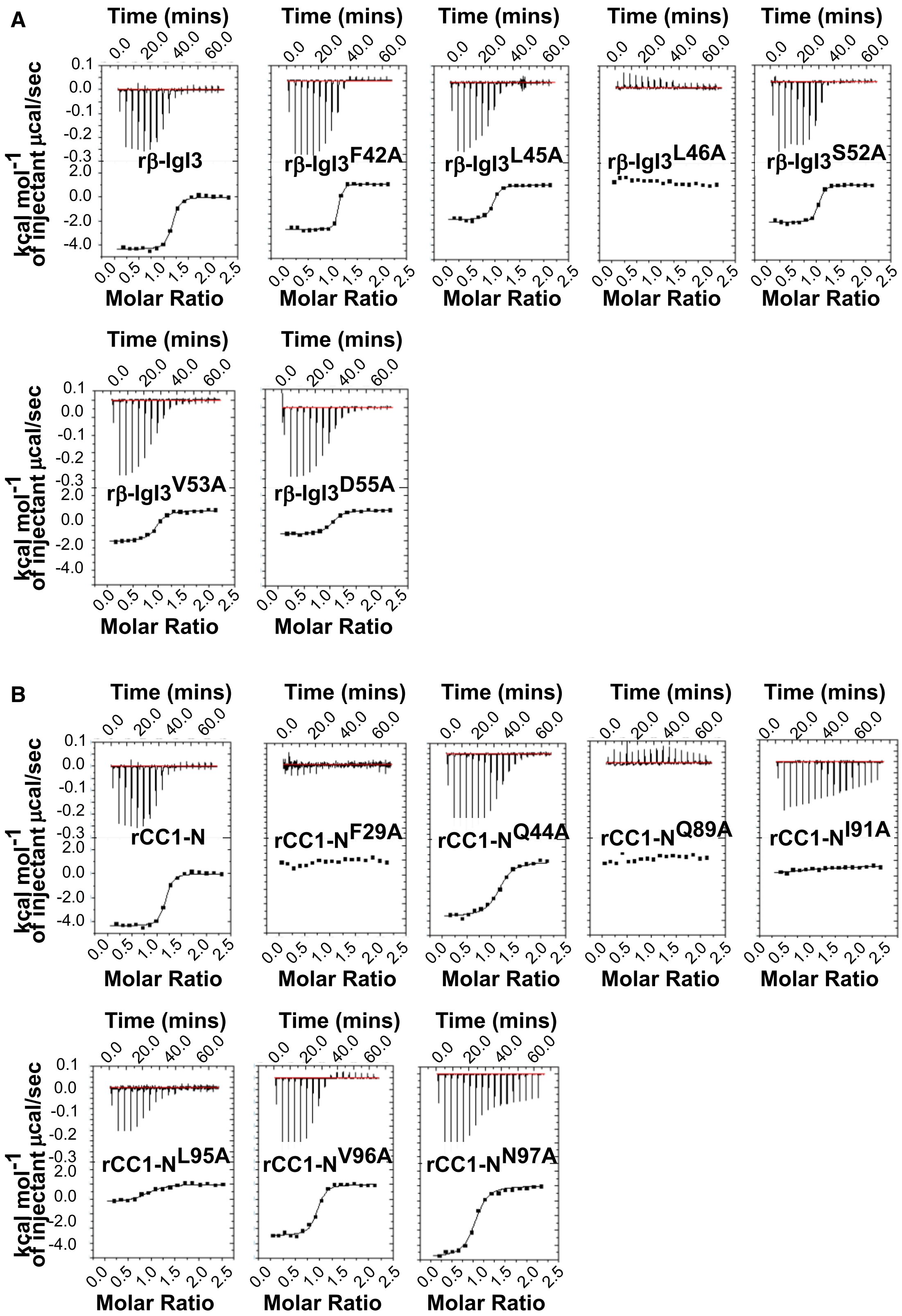

Figure EV4.

 © 2021 The Authors

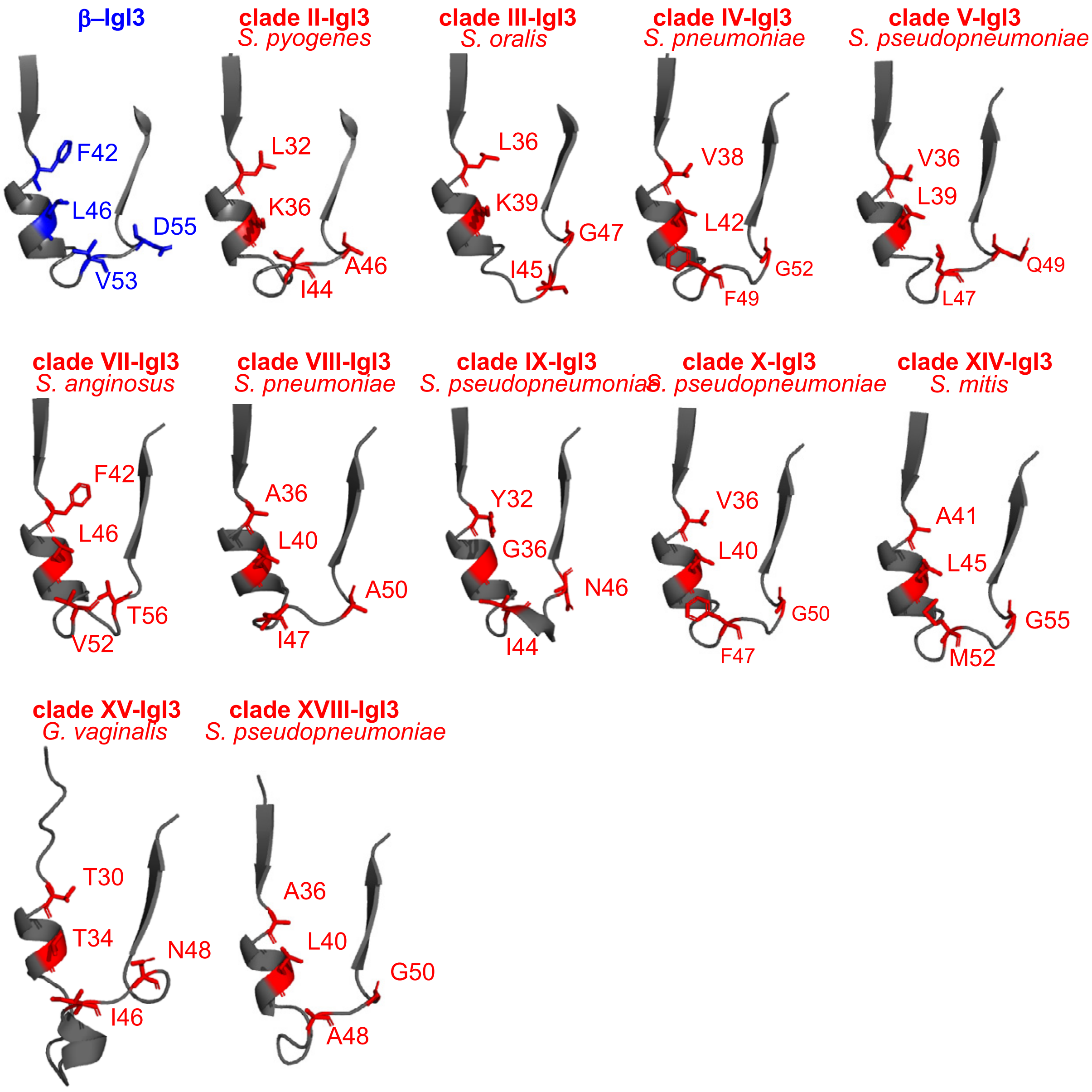

Figure EV5. Residues in β-IgI3 required for binding CEACAM1 are not conserved in IgI3 homologs.

Close-up of the *C* strand, α-helix and *D* strand of β-IgI3 and IgI3 homologs, indicating the amino acids present at residue sites in β-IgI3 that bind CEACAM1 (CC1). The clade and bacterial species of the sequence in each model is shown.
