# Supplementary figures and images for: Bacterial protein domains with a novel Ig‐like fold target human CEACAM receptors
Source: EMBO J. 2021 Feb 1;40(7):e106103. doi: 10.15252/embj.2020106103 (PMC8013792; doi:10.15252/embj.2020106103)

## STAIN

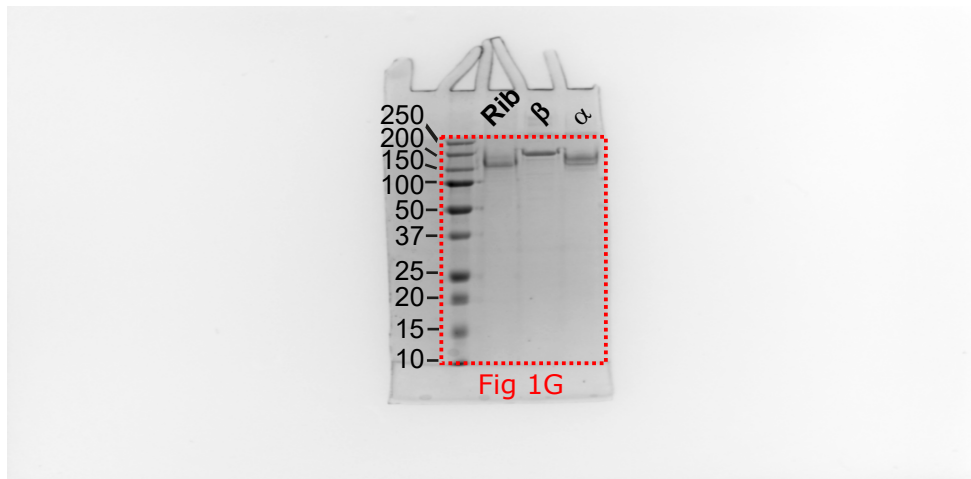

## BLOT

### PHOTOGRAPH

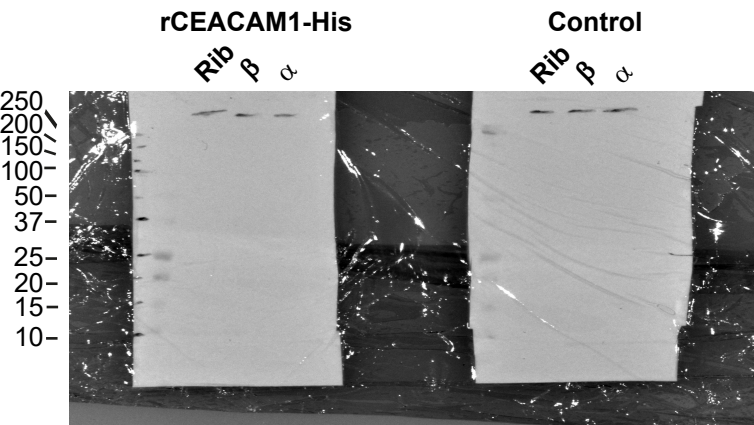

### CHEMILUMINESCENCE

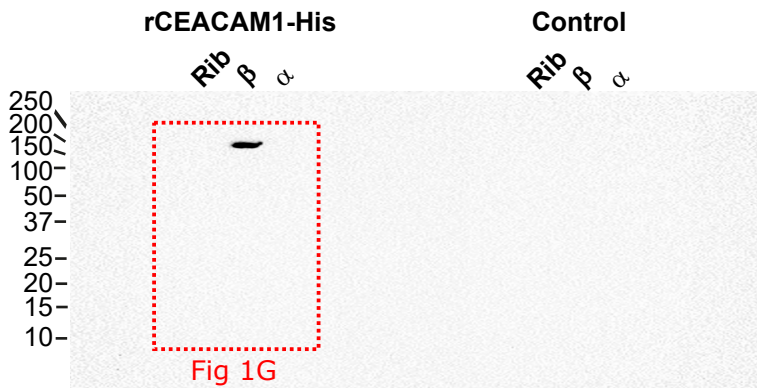

Supplement: Supplementary file 4 — Source Data for Figure 1 [file EMBJ-40-e106103-s001.pdf]

STAIN

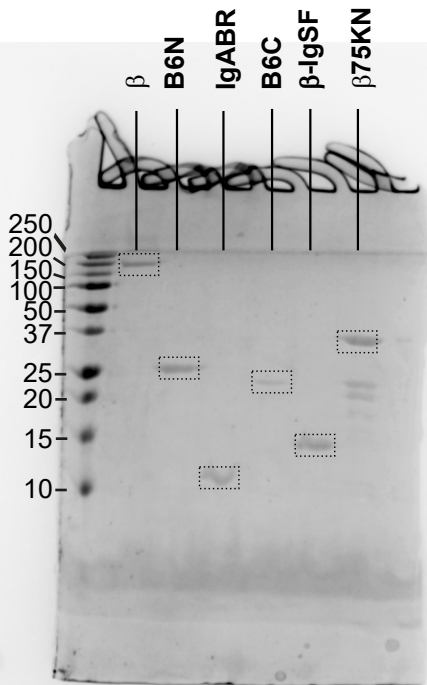

Supplement: Supplementary file 5 — Source Data for Figure 2 [file EMBJ-40-e106103-s003.pdf]
